# Functional Magnetic Resonance Imaging and Electroencephalography in Psychiatric Disease Diagnosis: A Review of New Findings and Comparisons

**DOI:** 10.7759/cureus.106048

**Published:** 2026-03-29

**Authors:** Maria Tsouka, Alexandra Gkolfaki, Efthalia Tzila, Eleni Panagouli

**Affiliations:** 1 Department of Psychology, Université Lumière Lyon 2, Lyon, FRA; 2 Department of Child and Adolescent Psychiatry, Peristeri Mental Health Center, 2nd Regional Health Authority, Regional Mental Health Services Network, Athens, GRC; 3 School of Medicine, National and Kapodistrian University of Athens, Athens, GRC; 4 Department of Basic Sciences, University of Nicosia, UNIC Athens, Athens, GRC

**Keywords:** anxiety disorders, attention-deficit hyperactivity disorder, electroencephalography, functional magnetic resonance imaging, major depressive disorder, schizophrenia

## Abstract

Psychiatric diagnosis continues to be grounded predominantly in symptom-based classificatory systems, most notably the Diagnostic and Statistical Manual of Mental Disorders and the International Classification of Diseases. Although these frameworks provide essential standardization and facilitate diagnostic reliability in clinical practice, they remain largely descriptive in nature and do not correspond directly to well-characterized neurobiological mechanisms. This limitation has stimulated increasing scientific interest in the identification of objective biomarkers that may enhance diagnostic precision, improve prognostic assessment, and support treatment stratification in psychiatric disorders. The present review synthesizes recent advances in the application of functional magnetic resonance imaging and electroencephalography for the purpose of psychiatric disease diagnosis.

Recent progress in computational psychiatry, together with developments in machine learning methodologies and the growing availability of large-scale neuroimaging datasets, has accelerated efforts to translate these approaches into clinically relevant diagnostic instruments. In particular, studies employing functional connectivity analyses have revealed reproducible patterns of large-scale network dysregulation across several psychiatric conditions, including major depressive disorder, schizophrenia, attention-deficit hyperactivity disorder, and anxiety disorders. Complementary electrophysiological research utilizing electroencephalography has likewise demonstrated alterations in neural oscillatory activity, event-related potentials, and microstate organization, collectively suggesting disruptions in the temporal coordination of neural dynamics in psychiatric populations.

Despite these advances, substantial methodological and translational challenges remain prior to the routine clinical implementation of these techniques. A considerable proportion of reported findings are derived from group-level analyses and exhibit limited generalizability at the level of individual patients. Moreover, methodological heterogeneity across studies, relatively small sample sizes, and the substantial diagnostic overlap that characterizes many psychiatric disorders continue to constrain the reliable clinical translation of neuroimaging-derived biomarkers.

## Introduction and background

Despite major advances in neuroscience and neuroimaging technologies, psychiatric diagnosis continues to rely primarily on symptom-based classification systems such as the Diagnostic and Statistical Manual of Mental Disorders and the International Classification of Diseases. These systems have substantially improved diagnostic reliability and communication across clinical settings. However, they remain fundamentally descriptive and do not necessarily correspond to discrete neurobiological mechanisms underlying psychiatric illness [1,2]. This limitation has motivated the search for biologically grounded approaches that can complement current diagnostic frameworks.

In this context, biomarkers, defined as objective, measurable indicators of biological processes, have attracted increasing interest in psychiatry. Biomarkers derived from neuroimaging and electrophysiological techniques have been proposed as tools for improving diagnostic accuracy, predicting treatment response, and enabling more precise patient stratification within heterogeneous psychiatric populations [3,4]. Such approaches are central to the emerging field of precision psychiatry, which aims to tailor diagnosis and treatment to individual neurobiological profiles.

Neuroimaging has become a key component of this effort, particularly with the use of functional magnetic resonance imaging (fMRI) and electroencephalography (EEG). These techniques provide complementary information about brain function. fMRI measures hemodynamic changes associated with neuronal activity, offering high spatial resolution and enabling the study of large-scale brain networks. In contrast, EEG directly records neuronal electrical activity with millisecond-level temporal resolution, capturing fast neural dynamics that are not accessible with hemodynamic methods. The integration of these modalities, often referred to as multimodal neuroimaging, is especially valuable, as it combines spatial and temporal information to provide a more comprehensive understanding of brain function.

Recent advances in computational psychiatry and machine learning have further expanded the potential of these techniques. Machine learning methods can analyze complex, high-dimensional neuroimaging data to identify patterns that may serve as diagnostic or prognostic indicators of psychiatric disorders [1,2]. By integrating multimodal data with computational models, researchers aim to move beyond descriptive classifications toward biologically informed systems.

Nevertheless, several challenges continue to limit the clinical translation of these approaches. Many neuroimaging studies rely on relatively small sample sizes, reducing statistical power and generalizability. In addition, the substantial heterogeneity of psychiatric disorders complicates the identification of disorder-specific neural signatures. Reported biomarkers often show limited reproducibility across independent cohorts, and findings based on group-level differences frequently lack sufficient accuracy when applied to individual patients. These limitations highlight the need for standardized methodologies, larger multicenter datasets, and rigorous validation strategies.

The present study summarizes recent developments in the application of fMRI and EEG to psychiatric disease diagnosis. Particular emphasis is placed on network-level models of psychopathology, machine learning approaches to diagnostic prediction, electrophysiological biomarkers, and emerging multimodal integration strategies. The comparative strengths and limitations of each modality are discussed, along with the key methodological and translational barriers that must be addressed before neuroimaging-based diagnostics can be incorporated into routine clinical practice.

## Review

Methods

Study Design

This literature review was conducted following the Preferred Reporting Items for Systematic Reviews and Meta-Analyses (PRISMA) guidelines to ensure transparency, reproducibility, and methodological rigor. The aim was to systematically identify and synthesize studies investigating the role of fMRI, EEG, and machine learning approaches in psychiatric diagnosis.

Literature Search Strategy

A comprehensive literature search was performed across three electronic databases: PubMed, Scopus, and Google Scholar. The search covered studies published between January 2010 and February 2026.

Search terms were developed to capture key concepts related to neuroimaging, electrophysiology, and psychiatric diagnosis. The following combinations of keywords and Boolean operators were used: “functional magnetic resonance imaging” OR “fMRI”, “electroencephalography” OR “EEG”, “psychiatric disorders” OR “mental disorders” OR specific diagnoses (e.g., “major depressive disorder”, “schizophrenia”, “bipolar disorder”, “attention-deficit/hyperactivity disorder”, “anxiety disorders”), AND “diagnosis”, “biomarkers”, “machine learning”, “classification”, OR “prediction”.

Reference lists of all included studies and relevant review articles were manually screened to identify additional eligible publications.

Eligibility Criteria

In order to be considered eligible, the studies should focus on at least one of the following psychiatric disorders: major depressive disorder, schizophrenia spectrum disorders, bipolar disorder, attention-deficit/hyperactivity disorder (ADHD), or anxiety disorders. Additionally, the use of fMRI, EEG, or combined (multimodal) neuroimaging approaches and investigation of diagnostic, prognostic, or biomarker-related outcomes should also be discussed. Only original research articles published in English and involving human participants were included. Case reports, editorials, conference abstracts, animal studies, or in vitro research and non-peer-reviewed articles were excluded. Moreover, studies not directly addressing psychiatric diagnosis or biomarker identification, focusing exclusively on neurological disorders (e.g., epilepsy, Alzheimer's disease) without psychiatric relevance, and lacking sufficient methodological detail or reporting incomplete results were not included. 

Study Selection

All retrieved records were screened in two stages. First, titles and abstracts were reviewed to exclude clearly irrelevant studies. Second, full-text articles were assessed for eligibility based on the predefined inclusion and exclusion criteria. Duplicates were removed prior to screening. 

Data Extraction

Data were systematically extracted using a standardized data extraction form. The following information was collected from each study: author(s) and year of publication, study population and sample size, psychiatric diagnosis, neuroimaging modality (fMRI, EEG, or multimodal), type of analysis (e.g., resting-state connectivity, task-based paradigms, electrophysiological measures, machine learning models), and main findings related to diagnostic or prognostic biomarkers.

Data Synthesis

Given the heterogeneity in study designs, imaging modalities, analytical approaches, and outcome measures, a qualitative synthesis was conducted. Particular emphasis was placed on identifying convergent findings, methodological limitations, and factors affecting reproducibility and clinical translation.

fMRI in psychiatric diagnosis

Network-Level Conceptualization of Psychopathology

Resting-state fMRI (rs-fMRI) has reshaped contemporary models of psychiatric disorders by emphasizing intrinsic connectivity patterns across large-scale brain networks. Rather than focal structural abnormalities, psychiatric conditions appear to reflect distributed network dysregulation. Notably, efforts to define biologically informed subtypes of major depressive disorder, so-called depression biotypes, have generated considerable interest. These initial studies combined resting-state connectivity with symptom dimensions using canonical correlation analysis and hierarchical clustering, reporting four subtypes associated with differential response to transcranial magnetic stimulation (TMS). However, independent replication attempts, including Dinga et al. [5], did not reproduce significant brain-symptom canonical correlations or stable clusters under stricter validation procedures and external samples. These findings highlight that fMRI-defined biotypes remain preliminary and not ready for clinical application, underscoring the need for larger multi-site datasets, rigorous statistical validation, and out-of-sample replication before subtyping can inform individualized treatment.

Major depressive disorder has been associated with hyperconnectivity within the default mode network (DMN), potentially underlying maladaptive rumination and self-referential processing [3]. Schizophrenia has been linked to aberrant salience network function and disrupted integration between executive and default networks [4]. ADHD demonstrates frontostriatal circuit dysfunction affecting inhibitory control [6]. Anxiety disorders, in contrast, involve the dysregulation of amygdala-prefrontal circuitry, which underlies impaired threat processing [7]. Convergent evidence from recent meta-analyses of psychophysiological interaction (PPI) studies and current circuit-level reviews indicates that key prefrontal regions, including the ventrolateral (vlPFC), dorsolateral (dlPFC), and dorsomedial prefrontal cortex (dmPFC), normally regulate amygdala reactivity. In anxiety disorders, these circuits are consistently disrupted, with amygdala hyperactivity coupled with reduced or inefficient prefrontal control, contributing to heightened and poorly regulated threat responses.

This network-based framework aligns with dimensional models of psychopathology and supports the hypothesis that psychiatric disorders are disorders of connectivity rather than localized pathology [4]. Meta-analytic evidence suggests that several psychiatric disorders share convergent structural and functional alterations within regions such as the anterior insula and dorsal anterior cingulate cortex, indicating the presence of partially shared neurobiological substrates across diagnostic categories [4].

Machine Learning and Predictive Modeling

Machine learning approaches have increasingly been applied to large neuroimaging datasets in an effort to develop predictive models capable of classifying psychiatric disorders or forecasting treatment outcomes. Supervised learning algorithms such as support vector machines, random forests, and deep neural networks have demonstrated promising classification performance in several psychiatric datasets. Under controlled experimental conditions, classification accuracies ranging from 70% to 90% have been reported in studies of schizophrenia and major depressive disorder [8-10].

However, these results should be interpreted with caution. Many studies rely on relatively small samples and cross-validation procedures that may inflate classification accuracy. Recent large-scale reproducibility analyses suggest that robust and generalizable brain-behavior associations often require datasets containing thousands of participants [11]. Consequently, while machine learning methods offer considerable promise, substantial methodological improvements and larger collaborative datasets will likely be required before such models can achieve reliable clinical applicability.

Methodological Considerations

The strengths of fMRI include high spatial resolution, whole-brain coverage, and access to subcortical structures critical in psychiatric pathology. However, limitations include low temporal resolution, high financial cost, motion sensitivity, and variability across acquisition sites. Furthermore, diagnostic heterogeneity complicates biomarker specificity.

EEG in psychiatric diagnosis

Oscillatory Dynamics and Quantitative EEG (qEEG)

EEG provides direct measurement of neuronal oscillations and is uniquely suited to examine fast temporal dynamics. qEEG has enabled frequency-domain analysis and functional connectivity mapping.

The theta/beta ratio in ADHD has been extensively studied [12]. However, its diagnostic specificity remains debated, and it is not currently used in clinical practice [12]. Frontal alpha asymmetry has been proposed as a biomarker in depression, yet replication has yielded inconsistent results [13]. Schizophrenia is characterized by impaired gamma-band synchrony, suggesting disrupted cortical integration [14].

These oscillatory abnormalities offer mechanistic insight into altered neural dynamics that are not accessible via hemodynamic imaging.

Event-Related Potentials and Microstates

Event-related potentials, particularly P300 and mismatch negativity (MMN), are among the most replicated electrophysiological abnormalities in schizophrenia [15]. These components index deficits in cognitive control and sensory processing.

EEG microstates, reflecting transient global brain states, have emerged as promising markers of large-scale network organization. Recent quantitative work shows that durations, occurrences, and coverage exhibit good to excellent test-retest reliability, whereas transition metrics have poor reliability and should be interpreted with caution. Altered microstate metrics have been observed in mood and anxiety disorders, but effect sizes are small and heterogeneous, limiting immediate diagnostic utility. By contrast, in the psychosis spectrum, deviations in classes C (often increased) and D (often decreased, particularly in unmedicated states) are more consistently observed. Overall, EEG microstates should be considered research biomarkers, rather than disorder-specific diagnostic tools, pending standardized pipelines and multicenter validation [16,17].

Artificial Intelligence and Clinical Applicability

Recent deep learning approaches applied to raw EEG signals have enhanced classification performance [18]. Importantly, EEG is portable, cost-effective, and suitable for repeated longitudinal assessments, making it particularly attractive for translational applications such as neurofeedback and closed-loop neuromodulation. Nevertheless, spatial localization limitations and susceptibility to artifacts constrain interpretability.

Comparative perspective and multimodal integration

EEG and fMRI provide fundamentally complementary information. fMRI delineates the spatial architecture of dysfunctional networks, whereas EEG captures rapid electrophysiological dynamics. Simultaneous EEG-fMRI acquisition has evolved substantially since foundational work [19], with recent methodological and analytical advances that improve acquisition, artifact correction, and preprocessing [20-22]. Notably, high-field 7 Tesla EEG-fMRI has been demonstrated with millisecond temporal and sub-millimeter spatial resolution [23], offering unmatched spatiotemporal insight into brain function. These developments have expanded applications, including simultaneous neurofeedback paradigms and cognitive neuroscience studies [24]. However, replication across studies is limited, and methodological heterogeneity remains significant; consequently, simultaneous EEG-fMRI is currently a research tool rather than a clinically validated diagnostic technique. However, despite these technological improvements, neither modality alone achieves the sensitivity and specificity required for definitive psychiatric diagnosis at the individual level [2].

Simultaneous EEG-fMRI acquisition integrates electrophysiological and hemodynamic signals, enhancing the spatiotemporal characterization of neural processes [19]. Within a precision psychiatry framework, multimodal integration, including neuroimaging, genetics, behavioral phenotyping, and computational modeling, may improve the stratification of heterogeneous diagnostic categories [25-28] (Table 1).

**Table 1:** Comparative characteristics of fMRI and EEG in psychiatric research fMRI measures hemodynamic changes associated with neuronal activity through BOLD signals, offering high spatial resolution for mapping large-scale brain networks [2,4]. EEG records direct electrical activity from neuronal populations with millisecond-level temporal resolution, facilitating the study of rapid neural dynamics and oscillatory processes [14,16]. The comparative parameters presented are synthesized from established neuroimaging and biomarker literature [19,22]. fMRI: functional magnetic resonance imaging; EEG: electroencephalography; BOLD: blood-oxygen-level dependent

Characteristic	fMRI	EEG
Signal type	Hemodynamic (BOLD signal reflecting blood oxygenation changes)	Direct electrical activity of neuronal populations
Temporal resolution	Low (seconds)	Very high (milliseconds)
Spatial resolution	High	Limited
Brain coverage	Whole brain including deep structures	Primarily cortical surface activity
Cost	High	Relatively low
Portability	Limited to specialized facilities	Portable systems available
Clinical accessibility	Limited clinical use	Widely used in clinical neurology
Typical psychiatric biomarkers	Functional connectivity networks, resting-state networks	Oscillatory rhythms, event-related potentials, microstates
Strengths	Network mapping, deep brain structures, spatial localization	Temporal dynamics, real-time neural activity
Limitations	Expensive, motion sensitive, low temporal resolution	Limited spatial localization, artifact susceptibility

In addition to neuroimaging and electrophysiological approaches, genetic studies are playing an increasingly important role in contemporary neuropsychiatric research. Large-scale genome-wide association studies (GWAS) have identified numerous genetic variants associated with psychiatric disorders, highlighting their highly polygenic and complex nature. Importantly, many of these genetic risk factors are shared across diagnostic categories, supporting the transdiagnostic models of psychopathology. The integration of genetic data with neuroimaging measures, often referred to as imaging genetics, offers a promising avenue for linking molecular mechanisms to brain function and behavior. Such approaches may enhance the identification of biologically meaningful subtypes and improve the predictive power of diagnostic models. Within the framework of precision psychiatry, combining genetic, neuroimaging, and computational data may ultimately facilitate the development of more accurate, biologically informed diagnostic systems and personalized treatment strategies [26-28].

Translational barriers and reproducibility

Significant obstacles impede clinical translation. Psychiatric disorders are phenotypically heterogeneous and may reflect overlapping neurobiological dimensions rather than discrete categories. Small sample sizes, methodological variability, and overfitting in predictive models limit reproducibility [11]. Standardized acquisition protocols and large collaborative consortia are necessary for biomarker validation.

Moreover, ethical considerations regarding biological labeling and potential stigmatization warrant careful consideration in biomarker implementation.

Although numerous candidate biomarkers have been identified across neuroimaging, genetic, and peripheral biological domains, their clinical predictive value remains uncertain. The relationship between biological signatures and psychiatric disease expression is often probabilistic rather than deterministic, reflecting complex interactions between neural, immune, endocrine, and environmental systems [29].

Discussion

The integration of fMRI and EEG into psychiatric research has provided unprecedented insights into the neurobiological substrates of mental disorders. rs-fMRI studies have consistently demonstrated that psychiatric conditions are best conceptualized as network-level disorders rather than focal lesions. For instance, major depressive disorder has traditionally been associated with hyperconnectivity within the DMN, particularly in relation to maladaptive rumination and self-referential processing [3,4]. However, contemporary meta-analytic evidence suggests a more complex and heterogeneous pattern of network dysfunction. Rather than a uniform increase in DMN connectivity, both increases and decreases within the DMN have been reported across studies. Importantly, alterations in between-network connectivity appear to be equally, if not more, relevant. These include increased coupling between the DMN and frontoparietal control network (FPN), as well as enhanced limbic-DMN interactions, alongside decreased connectivity between the DMN and somatomotor networks and reduced limbic-FPN coupling. In addition, altered interactions between the ventral and dorsal attention networks (VAN-DAN) have been observed, with some evidence suggesting that these changes may scale with illness duration [3,4].

ADHD and anxiety disorders similarly exhibit characteristic alterations in frontostriatal circuits and amygdala-prefrontal connectivity, respectively [6,7]. Recent meta‑analytic evidence shows that ADHD neurocircuitry extends beyond core frontostriatal circuits to include frontal‑parietal, temporal, insular, and cerebellar regions, as well as gray matter reductions in the corpus callosum [30]. Task-based activation likelihood estimation (ALE) meta-analysis further demonstrates hypoactivation during executive function tasks in the inferior frontal gyrus, supramarginal and inferior parietal gyri, caudate, occipital cortex, and cerebellum [31]. These findings support a multi-network control model of ADHD rather than a purely frontostriatal account and reinforce dimensional models of psychopathology, suggesting that dysregulated functional connectivity may underlie shared cognitive and affective dysfunctions across diagnostic categories.

Complementary evidence from EEG highlights the temporal dynamics of neural activity, providing mechanistic insights inaccessible via hemodynamic imaging. Abnormal oscillatory patterns, such as altered theta/beta ratios in ADHD, frontal alpha asymmetry in depression, and impaired gamma synchrony in schizophrenia, suggest disturbances in network coordination and cortical integration [12-14]. Event-related potentials, including P300 and mismatch negativity, further index deficits in cognitive control and sensory processing, while microstate analyses reveal disruptions in transient global brain states across mood and psychotic disorders [15-17]. The convergence between EEG-derived and fMRI-derived network disruptions underscores the potential utility of multimodal approaches in capturing complementary dimensions of psychiatric pathology.

Machine learning and computational psychiatry offer promising avenues to translate these findings into clinically actionable biomarkers. Predictive modeling using support vector machines, deep neural networks, and ensemble approaches has demonstrated diagnostic classification accuracies of 70-90% in controlled datasets [8,9]. Reported classification accuracies of 70-90% in small or single-site cohorts should be interpreted with caution. Converging evidence from large-scale methodological studies indicates that brain-behavior associations at the individual level are typically modest in effect size, making predictive models highly susceptible to overfitting and optimistic performance estimates, particularly in limited samples. Robust machine learning practice in neuroimaging, therefore, requires the use of leakage-safe, nested cross-validation frameworks, along with confound-aware evaluation strategies that account for factors such as age, sex, and site effects. In addition, multi-site datasets introduce variability related to scanner differences and acquisition protocols, necessitating appropriate harmonization techniques to ensure comparability across cohorts. Importantly, models that perform well under cross-validation often show reduced accuracy when applied to independent external datasets, highlighting the challenge of generalizability. Recent large-scale studies suggest that reliable and reproducible brain-behavior associations frequently require sample sizes in the order of hundreds to thousands of participants, particularly for low-to-moderate effect phenotypes. Consequently, future work should prioritize large collaborative datasets, standardized preprocessing pipelines, and rigorous external validation to establish clinically meaningful and generalizable predictive biomarkers.

Moreover, resting-state connectivity patterns and electrophysiological features have been explored as predictors of treatment response, with preliminary evidence suggesting that individualized prediction of antidepressant efficacy and symptom trajectories may be achievable [10,26-28]. While fMRI-based subtyping approaches, such as the "depression biotypes" proposed by Drysdale et al. [10], are conceptually promising, independent replication attempts (e.g., Dinga et al. [5]) have not confirmed the stability of canonical correlations or clustering solutions. These observations highlight the current preliminary nature of such biotypes and indicate that they are not yet suitable for guiding individualized treatment. Robust clinical translation will require large multi-site datasets, rigorous out-of-sample validation, and replication before fMRI-derived subtypes can be applied in practice. Nevertheless, several limitations impede clinical translation. Many studies rely on relatively small cohorts, and heterogeneity within and between diagnostic categories reduces the generalizability of models. Overfitting, methodological variability, and the absence of standardized acquisition protocols further constrain reproducibility [11].

Multimodal integration represents a critical strategy for addressing these limitations. Simultaneous EEG-fMRI acquisition allows for the joint assessment of electrophysiological and hemodynamic signals, improving spatiotemporal resolution and enabling more precise mapping of aberrant neural networks [19,27]. Beyond neuroimaging, integrating genetic, behavioral, and computational phenotyping data has the potential to refine patient stratification and uncover biologically informed subtypes within heterogeneous psychiatric populations [25,28]. Artificial intelligence-driven pipelines incorporating multimodal data are emerging as a powerful tool for developing individualized diagnostic and prognostic models, though widespread clinical application remains in its early stages.

Translational barriers remain substantial. Psychiatric disorders are phenotypically and neurobiologically heterogeneous, often reflecting overlapping symptom dimensions rather than discrete categories. Sample size limitations, cross-site variability, and inconsistent preprocessing pipelines contribute to challenges in replicating findings. Ethical considerations, including the risk of stigmatization and the implications of biologically informed diagnostic labeling, must also be carefully addressed as neuroimaging biomarkers move closer to clinical implementation. Large-scale consortia, standardized protocols, and open data-sharing initiatives are essential to enhance reproducibility, validate candidate biomarkers, and facilitate their translation into clinical practice [26-28].

In recent years, large-scale collaborative neuroimaging initiatives have further strengthened the network-based conceptualization of psychiatric disorders. Studies combining data from thousands of individuals across international cohorts have demonstrated that alterations in functional connectivity often transcend traditional diagnostic boundaries. Instead of discrete disease-specific signatures, many psychiatric conditions appear to share partially overlapping patterns of dysconnectivity involving the default mode, salience, and frontoparietal control networks. These findings support emerging transdiagnostic frameworks and suggest that future diagnostic systems may rely on biologically defined dimensions of brain function rather than purely symptom-based categories [29,32-34].

## Conclusions

fMRI and EEG have advanced the understanding of the neurobiology of psychiatric disorders by revealing abnormalities in brain connectivity, neural dynamics, and electrophysiological activity. However, their clinical application remains limited due to the poor reproducibility of biomarkers and the heterogeneity of psychiatric conditions. Progress will require larger datasets, standardized methods, and rigorous validation. In conclusion, integrating fMRI and EEG with computational modeling and multimodal approaches offers a promising framework for advancing precision psychiatry. While these methods currently remain investigational, ongoing efforts leveraging large-scale datasets, standardized methodologies, and artificial intelligence-driven analytics may ultimately enable the development of biologically informed, individualized diagnostic systems capable of improving both prognostic accuracy and treatment stratification. Future research should prioritize multimodal integration, longitudinal designs, and replication across diverse populations to ensure the robustness and generalizability of neuroimaging-based biomarkers in psychiatric disease diagnosis.
